# A Prospective Study on the Spectrum of Liver Abscess With Special Reference to Ruptured Abscess in a Tertiary Care Hospital

**DOI:** 10.7759/cureus.70743

**Published:** 2024-10-03

**Authors:** Amitayu Jana, Akash Kumar Ray, Snigdha Sarkar, Ujjwal Bhattacharya

**Affiliations:** 1 Urology, Institute of Post Graduate Medical Education & Research, Kolkata, IND; 2 General Surgery, Calcutta National Medical College and Hospital, Kolkata, IND

**Keywords:** amoebic liver abscess, ct (computed tomography) imaging, pair (percutaneous aspiration, percuatneous catheter drainage, : pyogenic liver abscesses

## Abstract

Introduction

Liver abscesses are one of the most concerning healthcare issues in Western countries, with a prevalence rate of three per 100,000. Although with the improvement in the socio-economic status and the health care system, its incidence has decreased in developed nations, pyogenic and amoebic liver abscesses are still high in resource-poor countries. Low socioeconomic conditions, improper hygiene, lack of awareness, and often a delay in diagnosis can lead to fatal complications and high mortality.

Methods

This prospective study was conducted in the Department of General Surgery, Calcutta National Medical College (CNMC) from 1st January 2019 to 31st August 2020 after obtaining approval from the Institute Ethical Committee (IRB no: EC-CNMC/2019/238/1). Patients over 10 years old with clinical and radiological features of liver abscesses were included in the study. Demographic, clinical, and treatment parameters were analyzed. Clinical and biochemical data were also compared statistically between ruptured and unruptured cases.

Results

Sixty patients with liver abscesses were included in the study. A pyogenic liver abscess (PLA) and amoebic liver abscess (ALA) were 28 and 32 cases, respectively. Most patients were between 21 and 40 years of age (53.3%, n=60), with male predominance (76.7%, n=60). The most common symptom was fever in both PLA (89.2%, n=28) and ALA (65.6%, n=32). Most abscesses were 5-10 cm in size (56.6%, n=60). Clinical parameters like pedal edema, ascites, respiratory distress, intercostal tenderness, and peritonitis were associated with ruptured abscesses. Biochemical parameters like low albumin raised total leucocyte count (TLC), increased prothrombin time, and large size of the abscess were predictors of ruptured abscess (p<0.001). Image-guided aspiration was performed in 14 patients (23.3%, n=60), and CT-guided percutaneous catheter drainage was done in 30 cases (50%, n=60). The most common organism isolated was *E. coli*. The ruptured abscess was diagnosed in six patients (10%, n=60). All ruptured abscesses required laparotomy and open surgical drainage. After three months of follow-up, a complete resolution of abscess cavities were seen in 38 patients (63.3%, n=60). Mortality in our study was 5 (8.3%, n=60).

Conclusion

Although modern diagnostic tools can efficiently diagnose liver abscesses, the identification of clinical features still has its place. Percutaneous catheter drainage is most commonly performed as a minimally invasive procedure without significant morbidity. Clinical suspicion of a ruptured liver abscess should be dealt without a delay. Improved hygiene and awareness can reduce its incidence, but early identification of clinical features, prompt diagnosis, and treatment can reduce mortality and morbidity.

## Introduction

Liver abscess is the most common type of visceral abscess of the body. The incidence is around three per 100,000 in Western countries. Although the mortality has decreased significantly over a few decades, the incidence still remains high [1]. It is classified into four categories: pyogenic liver abscess (PLA), amoebic liver abscess (ALA), mycobacterial and fungal abscess. However, PLA is more common in developed countries, whereas ALA is more prevalent in developing countries. Mycobacterial and fungal abscesses are extremely rare entities confined to immunosuppressed, cancer, and transplant recipients [2]. In the Indian subcontinent, Africa, Southeast Asia, and other developing countries where the incidence is still high, diagnosis is often delayed, leading to fatal complications.

Pyogenic liver abscesses are quite common in Western countries. In 1938, Ochsner and DeBakey first described the incidence of pyogenic liver abscess to be 8/100,000. Pitt and Zuidemia in 1975 reported 13/100,000 cases of hospital admission [3,4]. Diabetes and chronic kidney disease act as major risk factors for PLA. The sources are biliary (30-40%), portal (10-20%), hepatic (5-10%), traumatic (<5%) and rarely inflammatory spread from other adjacent organs.

Amoebic liver abscesses are more common in developing countries, particularly tropical zones, due to an increased incidence of amoebic colitis owing to poor sanitation and low socio-economic conditions. ALA is more prevalent in males.

Modern diagnostic tools can accurately diagnose liver abscesses, whereas serological testing discriminates between pyogenic and amoebic etiology. In the early days, conservative and open surgical treatment were the only options, where complications and mortality were an issue. Image-guided aspiration not only prevents rupture but also escapes complications of surgery [5]. However, in developing countries like India, the disease is more prevalent either due to a lack of awareness of proper hygiene and sanitation or a high incidence of amoebic colitis. Moreover, there appears to be a delayed consultation because of various socioeconomic factors and a lack of diagnostic tools in remote areas. These result more growing numbers of ruptured liver abscesses. Mortality of ruptured liver abscess is high (>20%). It is always an emergency, and laparoscopic or open surgical evacuation of pus is indicated [6,7]. However, the clinical picture of an impending rupture or already ruptured liver abscess is not adequately described in the literature. An early diagnosis and prompt intervention not only reduce the mortality but also minimize the duration of hospital stay.

We have documented a series of 60 cases of liver abscess with special reference to ruptured abscess to understand its clinical predictors, diagnosis, and various treatment options. We also have analyzed the number of patients suffering from liver abscesses that progressed to rupture along with early symptoms and signs of an impending or ruptured liver abscess and intervention to reduce the mortality.

## Materials and methods

This prospective study was conducted in the Department of General Surgery, Calcutta National Medical College (CNMC), Kolkata, after obtaining approval from the Institute Ethical Committee (IRB no: EC-CNMC/2019/238/1) from 1st January 2019 to 31st August 2020.

Selection of cases

Patients aged more than 10 years who attended surgical OPD and emergency department of Calcutta National Medical College, Kolkata, with clinical and radiological features of liver abscess, liver abscess with clinical features of impending rupture or already ruptured abscess were included in the study.

Patients aged less than 10 years, infected hydatid cysts, hepatocellular carcinoma with secondary abscess, and who did not want to be a part of the study were excluded.

Study protocol

Data collection was started after explaining the purpose of the study and obtaining informed consent from the patients. Information regarding history, careful clinical examination, appropriate radiological, and hematological investigation, and operative interventions were recorded into a pretested proforma.

A trial of empirical antibiotics (third-generation cephalosporin and metronidazole) was prescribed to patients with multiple small abscesses, a single small abscess without any features of toxemia. On getting the report of amoebic serology, if it was negative, we continued with parenteral antibiotics. Image-guided aspiration was performed in patients with large abscesses (>5 cm), symptoms that persist with antibiotics, or any signs of impending rupture. The aspirated pus was sent for culture and sensitivity. Response to treatment was evaluated by a decrease in fever, leucocytosis, and clinical improvement.

Indications to proceed to CT-guided percutaneous catheter drainage were the persistence of sepsis, worsening of clinical features, failure to improve after a reasonable period, failed percutaneous aspiration, or thick abscess contents [8]. Contraindications to percutaneous catheter drainage included coagulopathy, the lack of a safe or appropriate access route, and multiple macroscopic abscesses with intervening septa and ascites.

Operative drainage was indicated for patients with ruptured abscesses, failed or contraindications to percutaneous drainage and patients who required laparotomy for clinical suspicion of other abdominal pathology. Drain amount was measured daily for both percutaneous drainage and laparotomy. The drain was removed if the output of content was less than 30 ml for 24 hours. If serology was positive for amoeba, then metronidazole remained the drug of choice as it is highly effective, inexpensive, and has the advantage of being effective for intestinal as well as extra-intestinal amoebiasis. The dose regimen is 750 mg three times daily for 10 days for adults and 30-50 mg/kg/day for pediatric patients.

All patients were advised with oral antibiotics for two weeks post-discharge. Patients were followed up with ultrasonography after three months to determine the resolution of the abscess cavity.

Statistical analysis

For statistical analysis, data were entered into a Microsoft Excel spreadsheet and then analyzed by SPSS software (version 27.0; IBM Corp., Armonk, NY) and Graph Pad Prism version 5 (GraphPad Software, Boston, MA). Data are represented in descriptive form with a 95% confidence interval (CI). Fisher's exact test and Wilcoxon-Mann-Whitney U test were performed to determine statistical significance between clinicopathological variables with ruptured liver abscess. P value<0.05 was considered statistically significant.

## Results

Among 60 cases, the amoebic liver abscess was 32 (53.3%, n=60), and the pyogenic liver abscess was 28 (46.7%, n=60). Incidence of both PLA and ALA was predominantly observed between 21-40 years (53.3%, n=60). Only 4 (6.7%, n=60) patients were older than 60 years in our study. Of the total 28 cases of PLA, the maximum number of patients were seen in age groups 21-40 years and 41-60 years (42.8%, n=28). Gender distribution shows both PLA (85.7%, n=28) and ALA (68.75%, n=32) were more common among male patients (Table 1).

**Table 1:** Distribution of liver abscess cases according to demographic data. Data presented as percentage. PLA: pyogenic liver abscess, ALA: amoebic liver abscess.

Demographic data	PLA (%)	ALA (%)
Gender		
Male	24 (85.7)	22 (68.7)
Female	4 (14.2)	10 (31.2)
Age (years)		
21-40	12 (42.8)	20 (62.5)
41-60	12 (42.8)	12 (37.5)
>60	4 (14.4)	0 (0)
Total, N=60	N=28	N=32

**Figure 1:**
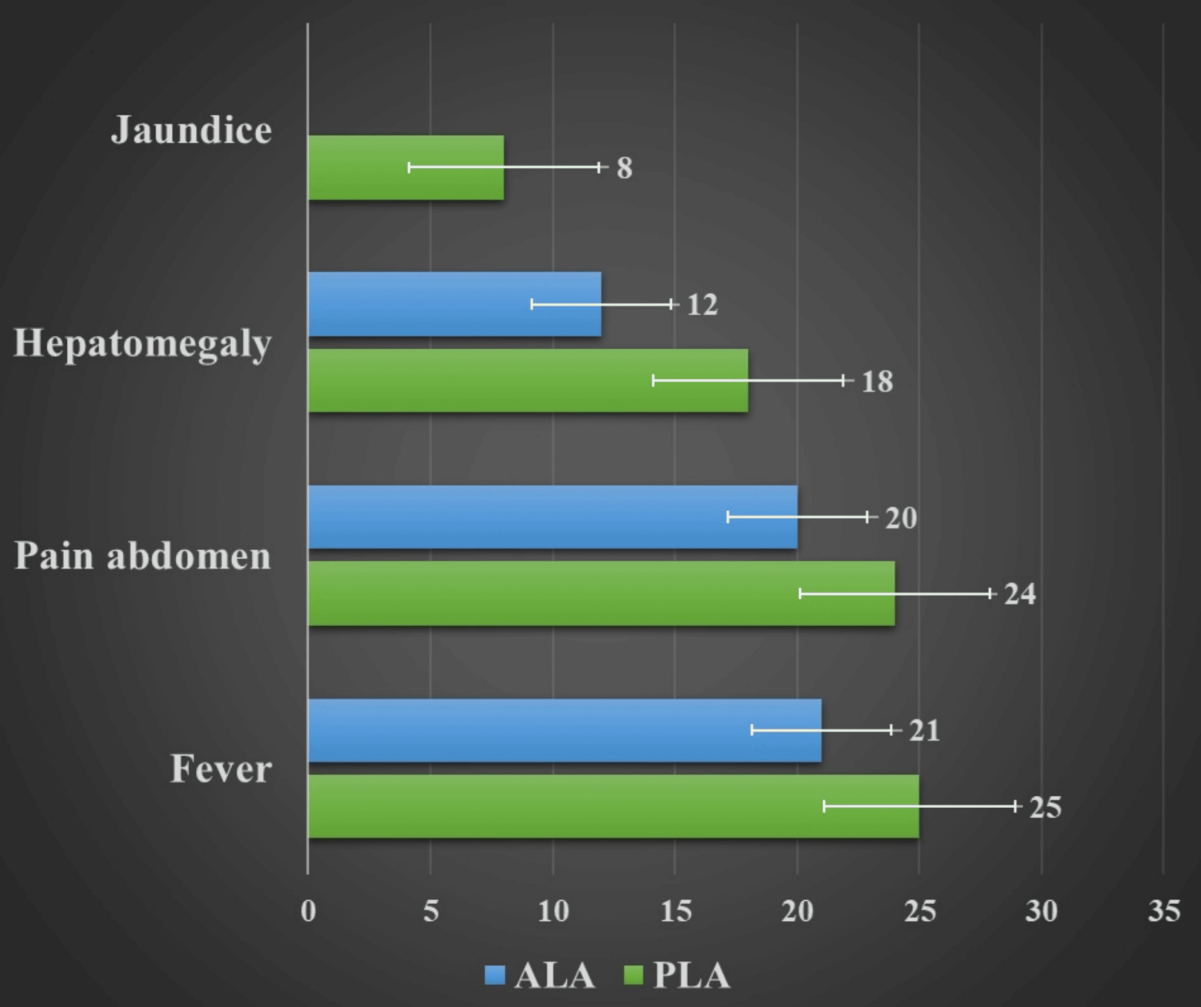
Clinical features of liver abscess cases. Data presented in numbers. PLA: pyogenic liver abscess, ALA: amoebic liver abscess.

Fever was the predominant symptom among 25 PLA (89.2%, n=28) patients and 21 ALA (65.56%, n=32) patients, followed by pain in the abdomen. Clinically, hepatomegaly was present in 18 patients with PLA (64.2%, n=28) and 12 patients with ALA (37.5%, n=32). Eight patients with PLA (28.5%, n=28) had jaundice (Figure 1). 

**Figure 2:**
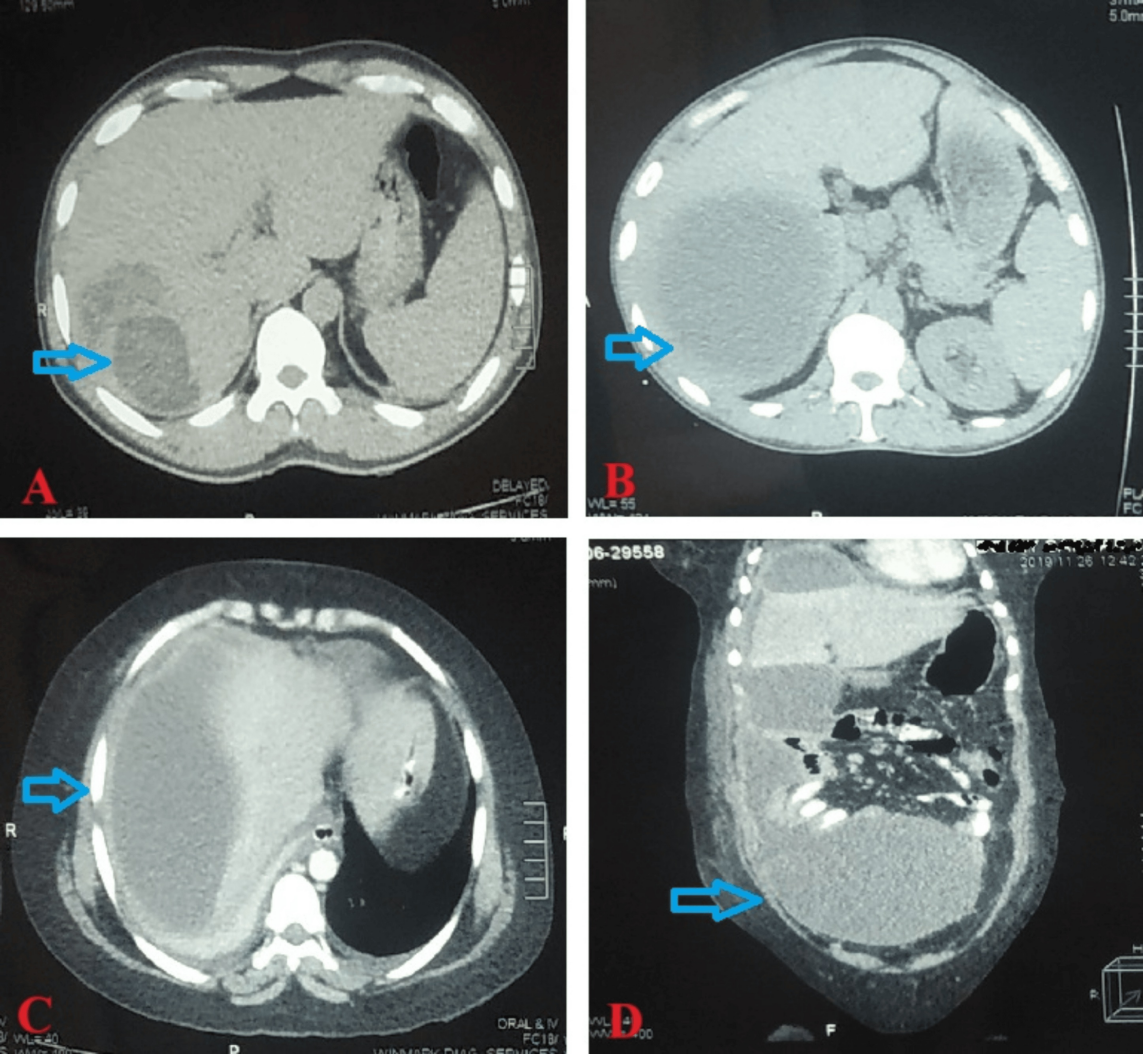
CT images of different liver abscess. (A) Small abscess (<5 cm) (axial section); (B) abscess between 5-10 cm (axial section); (C) large abscess (>10 cm) (axial section); (D) ruptured abscess with peritoneal dissemination (pointed arrow) (coronal section).

The CT image showed the size of the abscess cavity was highest in between 5 and 10 cm. Single and multiple abscesses were detected in 39 ((65%, n=60), 95% CI: 51.5-76.6) and 21 ((35%, n=60) 95% CI: 23.4-48.5) patients, respectively (Table 2) (Figures 2A-2C).

**Table 2:** Distribution of size of the abscess. Data represented as percentage, N=60. PLA: pyogenic liver abscess, ALA: amoebic liver abscess.

Size of abscess (cm)	PLA (%)	ALA (%)	Total (%)
<5	9 (32.1)	5 (15.6)	14 (23.3)
5-10	14 (50)	20 (62.5)	34 (56.6)
>10	5 (17.8)	7 (21.8)	12 (20)
Total	28 (100)	32 (100)	60 (100)

Among all the patients with liver abscesses 6 (10%, n=60) had presented with ruptured abscesses (Table 3) (Figure 2D).

**Table 3:** Outcome of liver abscess. Data represented as percentage, N=60.

Outcome	No. of patients (%)
Abscess (ruptured)	6 (10%)
Abscess (intact)	54 (90%)

Fischer’s exact test was performed to determine the strength of the association of various clinical parameters between patients with ruptured and unruptured liver abscesses. Incidence of specific signs like pedal edema (p<0.001, OR: 62.5), ascites (p<0.001, OR 85), respiratory distress (p<0.001, OR: 10.9), intercostal tenderness (p<0.001, OR: 40), and peritonitis (p<0.001, OR: 106) were found to be more prevalent in ruptured abscesses and associations were statistically significant (Table 4).

**Table 4:** Comparison of clinical features between ruptured and unruptured cases. Fisher's exact test performed. P-value<0.05 was considered statistically significant.

Clinical features	Rupture		Fisher's exact test		Odds ratio (95% CI)
	Yes (n=6) (%)	No (n=54) (%)	P-value	χ^2^	
Pain	4 (66.7)	40 (74.1)	0.634	0.247	0.63 (0.1-3.87)
Fever	6 (100)	40 (74.1)	0.320	2.029	4.65 (0.25-7.89)
Jaundice	1 (16.7)	7 (13)	1.000	0.064	1.34 (0.14-13.25)
Hepatomegaly	4 (66.7)	26 (48.1)	0.671	0.741	2.15 (0.36-12.76)
Pedal edema	5 (83.3)	4 (7.4)	<0.001	24.415	62.5 (5.81-72.83)
Ascites	5 (83.3)	3 (5.6)	<0.001	28.269	85 (7.39-97.76)
Respiratory distress	3 (50)	0 (0)	<0.001	28.421	10.9 (4.65-25.54)
Intercostal tenderness	5 (83.3)	6 (11.1)	<0.001	18.813	40 (3.98-402.45)
Peritonitis	4 (66.7)	1 (1.9%)	<0.001	29.697	106 (7.82-143.79)

**Figure 3:**
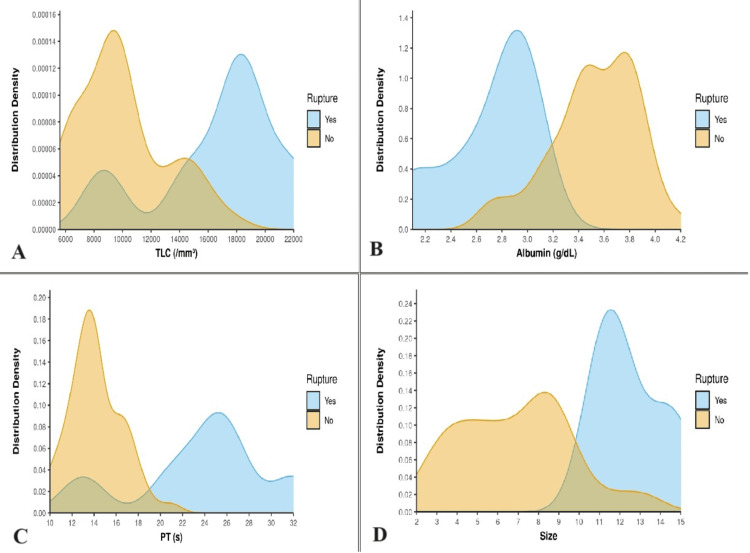
Distribution density of various risk factors of ruptured abscess. (A) Leucocyte count (TLC) (mm^3^); (B) albumin (g/dL); (C) prothrombin time (PT) (s); and (D) size of abscess cavity (cm).

Wilcoxon-Mann-Whitney U test was done to identify biochemical risk factors for ruptured abscess. Laboratory results were compared between two groups. Biochemical data suggests low mean hemoglobin (9.2±0.63 g/dL vs 10.46±1.34 g/dL, p=0.004), low albumin (2.72±0.35 g/dL vs 3.52±0.34 g/dL, p<0.001), increased prothrombin time (23.37±6.35s vs 14.15±2.34s, p=0.002) in ruptured abscesses. Raised leucocyte count (16733±4577 vs 10008±3067, p=0.006) and larger abscess size (12.5±1.64 cm vs 6.84±2.75 cm, p<0.001) was also predominant in ruptured cases (Table 5) (Figures 3A-3D).

**Table 5:** Comparison of laboratory parameters and abscess size between ruptured and unruptured cases. Wilcoxon-Mann-Whitney U test performed. P-value<0.05 was considered statistically significant. Data presented as mean±standard deviation.

Investigation	Rupture		Wilcoxon-Mann-Whitney U test	
	Yes (n=6) (%)	No (n=54) (%)	p-value	W
Hemoglobin (g/dL)	92.8±0.63	10.46±1.34	0.004	45
Leucocyte count (mm^3^)	16733±4577	10008±3067	0.003	282
Albumin (g/dL)	2.72±0.35	3.52±0.34	<0.001	15
Prothrombin time (s)	23.37±6.35	14.15±2.34	0.002	286
Size of abscess on largest diameter (cm)	12.5±1.64	6.84±2.75	<0.001	310

**Figure 4:**
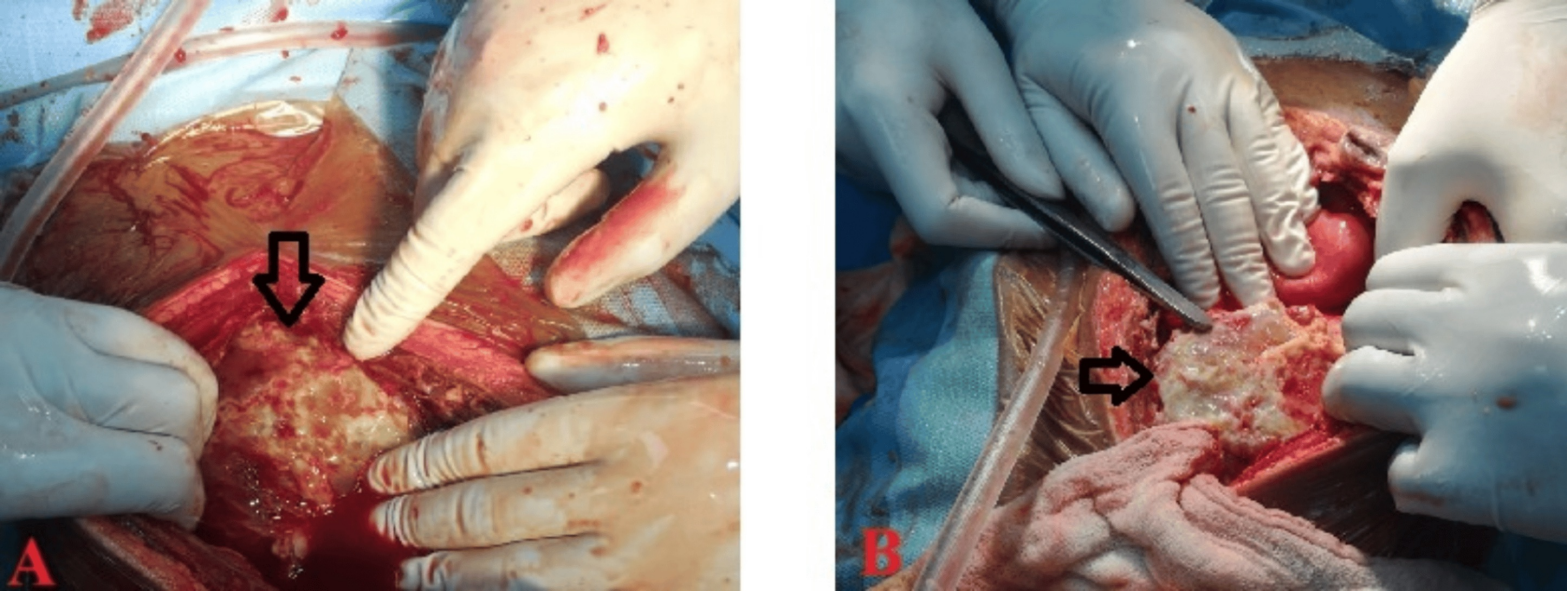
Open surgical drainage of liver abscess. (A) and (B) Intraoperative images of laparotomy for ruptured liver abscess with (slough in the pointed area) undergoing open drainage.

CT-guided percutaneous catheter drainage was the most commonly performed procedure for both PLA (53.5%, n=28) and ALA (46.8%, n=32). Conservative management with antibiotics alone were sufficient in 10 patients (16.6%, n=60). Open surgical drainage was performed in 6 (10%, n=60) cases (Table 6) (Figures 4A, 4B).

**Table 6:** Distribution of different management of liver abscess. Data presented in percentage with 95% CI. PLA: pyogenic liver abscess, ALA: amoebic liver abscess.

Management	PLA (%)	ALA (%)	Total (%)	95% CI
Conservative (only antibiotic)	4 (14.2)	6 (18.7)	10 (16.6)	13.8%-36.3%
Image guided aspiration with antibiotics	5 (17.8)	9 (28.1)	14 (23.3)	15.1%-38.1%
Image guided catheter drainage with antibiotics	15 (53.5)	15 (46.8)	30 (50)	29.3%-55.1%
Open surgical drainage with antibiotics	4 (14.2)	2 (6.2)	6 (10)	4.1%-21.2%
Total (N=60)	28 (100)	32 (100)	60 (100)	-

The most common cultured organism from PLA was *E. coli *(57.1%, n=28), followed by Klebsiella (21.4%, n=28) and *E. faecalis* (14.2%, n=28), *S. aureus *(7.1%, n=28) in PLA patients. Pus culture of ALA patients has revealed no growth (Table 7).

**Table 7:** Organism cultured in pyogenic liver abscess. Data represented as percentage, N=28.

Organism	No. of patient (%)
E. coli	16 (57.1)
K. pneumoniae	6 (21.4)
S. aureus	2 (7.1)
E. faecalis	4 (14.2)

**Figure 5:**
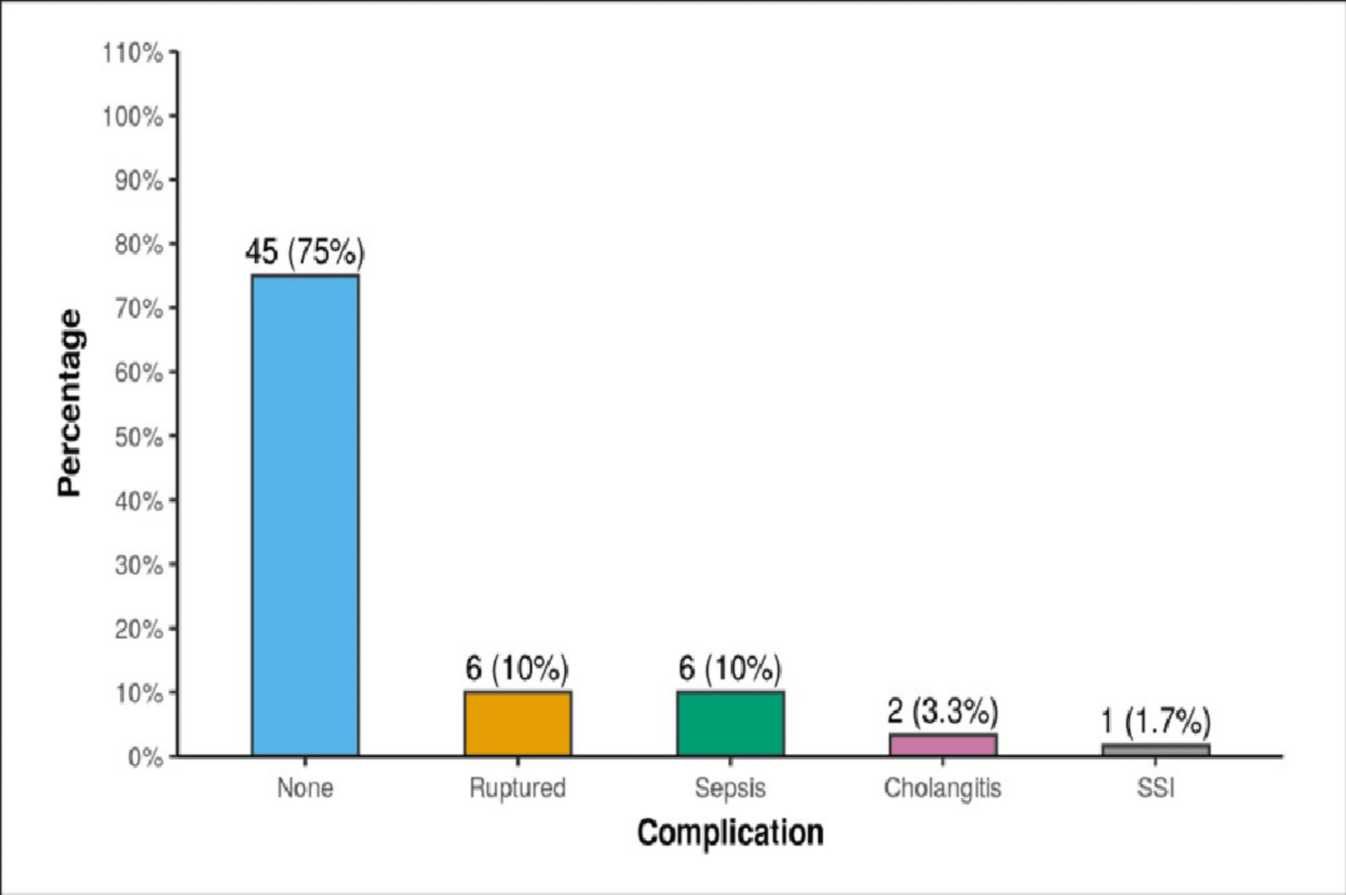
Complications of liver abscess. SSI: surgical site infection.

In the study population, 45 patients (75%, n=60) had no post-procedure complication. Among the rest of the patients, six patients (10%, n=60) had ruptured abscesses; six patients (10%, n=60) complicated by sepsis. Cholangitis and surgical site infection (SSI) were detected in 2 (3.3%, n=60) and 1 (1.7%, n=60), respectively (Figure 5).

**Figure 6:**
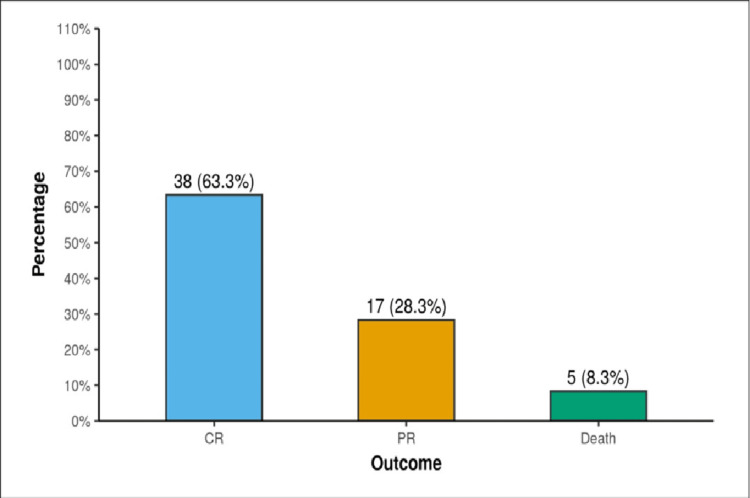
Outcome of liver abscess after three months of follow-up. CR: complete resolution; PR: partial resolution.

After three months of follow-up with ultrasonography, complete resolution of the abscess cavity was noted in 38 patients (63.3%, n=60), of which 12 cases were PLA (42.8%, n=60) and 26 cases were ALA (81.2%, n=32). Mortality in our study was 5 (8.3%, n=60) (Figure 6).

## Discussion

Our study had increased numbers of amoebic over pyogenic abscesses. Among 60 cases of liver abscess, ALA was 32 (53.3%) and PLA was 28 (46.7%). In developed countries, pyogenic abscesses were more common [9]. Due to poor socioeconomic reasons, the amoebic liver abscess is still more prevalent in India. None of the cases of liver abscess in this study came to be tubercular or fungal liver abscess.

In the present study, the highest incidence of liver abscess was noted between the second and sixth decade. The lowest incidence was noted for more than 60 years (6.7%, n=60). The incidence of abscess was higher between 21 and 40 years (53.3%, n=60), followed by 41 and 60 years (40.0%, n=60). Incidence of ALA is higher in the 21-40 year group compared to PLA. The incidence of ALA and PLA was similar in 41-60 years. The mean age in our series was 41.6 years, which matches with another Indian study by Ghosh et al., who reported a mean age of 40.5 years [10].

The most common presenting complaints among liver abscess patients were fever and abdominal pain. Agu et al. have also shown that in 91.3% of patients with ALA fever and abdominal pain were the common presentations [11]. Singh et al. also showed that 88% of study patients have fever as the most common presenting symptom [12].

Abscesses were limited to the right lobe in 50 (83.3%, n=60), the left lobe in 8 (13.3%, n=60), and both lobes in 2 (3.3%, n=60) of cases. The majority of the abscess cavities were between 5 and 10 cm in size in both ALA and PLA. According to Rajak et al., solitary abscesses were seen in 72% of cases and multiple abscesses were seen in 18%. Abscesses were located in the right lobe in 72% of cases and the left lobe in 12% of cases. Both lobe involvements were seen in 16% of their study [8].

Pedal edema (p<0.001, OR: 62.5), ascites (p<0.001, OR: 85), respiratory distress (p<0.001, OR: 10.9), intercostal tenderness (p<0.001, OR: 40), peritonitis (p<0.001, OR: 106) were determined to be strong clinical predictors of ruptured abscess. Biochemical parameters suggested that raised leucocyte count, increased prothrombin time, and low serum albumin level were observed in ruptured cases (p<0.001). Abscess size was also an independent risk factor for rupture (p<0.001) [13].

Surgical drainage of liver abscesses has been an accepted therapy for decades. The diagnosis and treatment of liver abscesses have changed due to advances in image-guided minimally invasive techniques. In the present study of 60 cases, conservative management was done on 10 (16.6%, n=60) cases. Amoebic serology was routinely performed. A study by McGarr et al. documented that 150 of 178 patients were managed successfully with drug therapy alone, with those demonstrating clinical deterioration or no improvement after 48 to 72 h of conservative management received percutaneous ultrasound-guided aspiration [14]. A 12 Fr pigtail catheter (Uromed™ PCN Catheter, Manish Medi Innovation, Bangalore, India) was used under ultrasound or CT guidance. No complications were noted due to this procedure, apart from local pain, which soon subsided after analgesics. Patients showed dramatic improvements in their symptoms and signs within 48-72 hours of the aspiration. Image-guided aspiration was done in 14 patients (23.3%, n=60), and percutaneous drainage in 30 patients (50%, n=60). According to Giorgio et al., percutaneous needle aspiration is an efficient, effective, and low-cost technique that can even be performed on an outpatient basis [15]. It is safe and free from complications. Zafar et al. opined that percutaneous needle aspiration is a safe, rapid, and effective method of treating liver abscesses. Routine aspiration is not indicated. It should be the initial line of treatment in abscess >300 cc, impending rupture, or abscess that do not respond to chemotherapy [16]. Laparotomy was performed in six cases of liver abscess (10%). Five cases presented as acute abdomen with peritonitis and respiratory distress, and the other case had multiple liver abscesses, which failed to respond after 48 hours of conservative management. On laparotomy, thorough peritoneal lavage was performed, and drains were kept for five days or until drain output was <30 ml for 24 hours. Thus, in the majority of cases, image-guided percutaneous aspiration/catheter drainage was the main form of surgical therapy. Antibiotics were continued in all patients for 14 days along with the percutaneous procedure. Today, laparoscopic drainage of liver abscesses is also thought to be a better alternative for patients with ruptured abscesses, failure or contraindication to percutaneous drainage, and thick or multiseptated abscesses [7]. Although the laparoscopic approach is associated with low morbidity and mortality, the lack of facilities for emergency laparoscopy and the availability of skilled laparoscopic surgeons limit its use. The majority of patients responded excellently to percutaneous aspiration/catheter drainage and antimicrobials.

Pus culture was positive in 28 patients. The most common cultured organism from PLA was *E. coli, *followed by Klebsiella and *E. faecalis*, *S. aureus*. Pus culture of ALA patients has revealed no growth. This negative result in both pus and blood culture might be due to prior antibiotic treatment. This is as per the studies conducted by Rahimian et al. and Johannsen et al., who also reported *E. coli* and Klebsiella to be the most common organisms cultured [17,18].

During treatment, 45 patients (75%, n=60) had no post-procedural complication. Apart from 6 (10%, n=60) ruptured abscesses, sepsis in six cases (10%, n=60), cholangitis in two cases (3.3%, n=60), and surgical site infection after laparotomy was diagnosed in one case (1.7%, n=60).

After follow-up for three months with ultrasonography, the majority of patients had complete radiological resolution (63.3%, n=60). Mortality in PLA and ALA were 3 (10.7%, n=28) and 2 (6.25%, n=32), respectively. All of them had undergone emergency laparotomies for ruptured liver abscesses.

Therefore, the clinical spectrum of liver abscess may vary from general symptoms like fever and pain in the abdomen to specific features like pedal edema, intercostal tenderness, respiratory distress, ascites, and peritonitis. Laboratory investigations also play an important role in clinical decision-making towards the identification of the severity of the abscess. Conglomeration of these clinico-pathological parameters not only help us to use minimally invasive image guided intervention but also guide us for prompt intervention for ruptured abscess. It is extremely important to recognize, manage and intervene liver abscess patients in a tertiary care hospital to prevent morbidity and mortality.

The main limitation of our study is the small sample size. It is a single-center prospective study. Anaerobic culture was not done due to a lack of facilities and financial obstacles. Lastly, Infrastructure for emergency laparoscopic drainage could also reduce mortality.

## Conclusions

Apart from fever, abdominal pain as general symptoms, specific clinical features like pedal edema, ascites, intercostal tenderness, respiratory distress, peritonitis and laboratory parameters like raised leucocyte count, hypoalbuminemia, prolonged prothrombin time, large abscess cavity are often predictors of an impending rupture. Image-guided percutaneous catheter drainage is a safe and most commonly performed minimally invasive procedure. It is imperative to identify ruptured abscesses at the earliest in a tertiary care hospital for prompt surgical drainage. This inference will help other clinicians to suspect and manage liver abscesses more frequently in the future. After detection, prompt intervention with image-guided aspiration or drainage can save many lives. A specific study of the ruptured cases helped us to study the prognostic parameters for rupture. Early detection of those parameters in patients will help clinicians to decide the urgency of intervention instead of conservative management. Therefore, the evaluation of liver abscess with all its spectrum from general to specific clinical features, treatment, and early identification of risk factors have paramount importance to prevent morbidity and mortality. 
